# Analytical and Molecular Recognition Strategies for Chinese Lacquerware Conservation

**DOI:** 10.3390/polym18121454

**Published:** 2026-06-10

**Authors:** Yuanyuan Liu, Yujia Liu, Xinhao Feng, Xinyou Liu

**Affiliations:** 1 College of Art, Huangshan University, Huangshan 245021, China; 310104@hsu.edu.cn; 2 College of Furnishing and Industrial Design, Nanjing Forestry University, Nanjing 210037, China; liuyujia@njfu.edu.cn (Y.L.); fengxinhao@njfu.edu.cn (X.F.)

**Keywords:** Chinese lacquerware, lacquer characterization, pyrolysis gas chromatography mass spectrometry, immunological identification, cultural heritage conservation

## Abstract

Chinese lacquerware is a multi-layered natural polymer composite whose characterization is complicated by burial degradation, organic–inorganic mixing, and the overlap of signals from lacquer, drying oils, proteins, polysaccharides, waxes, and pigments. This review evaluates analytical strategies for Chinese lacquerware by distinguishing three complementary levels of evidence: morphological and elemental observation, chemically specific molecular fingerprinting, and biomolecular source recognition. Microscopy, Fourier transform infrared spectroscopy (FTIR), Raman spectroscopy, and scanning electron microscopy–energy dispersive spectroscopy (SEM-EDS) are useful for identifying stratigraphy, pigments, fillers, and functional groups, but they are often insufficient for assigning degraded organic matrices and trace additives independently. Pyrolysis–gas chromatography/mass spectrometry provides more specific molecular evidence through diagnostic marker classes, including alkyl catechols, alkyl phenols, nitrogen-containing pyrolysis products, anhydrosugars, long-chain aliphatics, aldehydes, and ketones. Immunological assays based on lacquer glycoproteins further complement chemical analysis by supporting biological source differentiation, although their reliability depends on protein preservation, extraction efficiency, and antibody specificity. Representative case studies, including a seventeenth-century Swedish lacquered pipe, the Nanyue Kingdom lacquered ear cup, and a Tang Dynasty lacquered leather artifact, show that robust interpretation requires cross-validation among stratigraphic, elemental, spectroscopic, chromatographic, immunological, and archaeological evidence. The review concludes that integrated analytical workflows can improve material identification, clarify manufacturing sequences, assess degradation uncertainty, and provide more reliable evidence for conservation decision-making and the reconstruction of historical lacquer craftsmanship.

## 1. Introduction

Lacquerware constitutes a foundational material heritage of Chinese civilization, with origins stretching back to the late Neolithic Age, making it one of the earliest known coated materials in human history [1]. Over millennia, Chinese lacquerware evolved into a highly sophisticated production system with deep aesthetic, technological, and social significance, reflecting complex interactions among craft traditions, natural resources, regional cultures, and material technologies. Beyond its artistic value, lacquerware preserves material evidence of layered construction, pigment selection, organic additives, surface decoration, and historical coating practices. These features make lacquer artifacts important sources for reconstructing ancient craftsmanship, resource use, cultural exchange, and the historical transformation of natural polymer materials [1,2,3].

However, the scientific characterization of lacquerware remains challenging because ancient lacquer films are typically multi-layered, multi-component, and highly degraded. Their binding matrices may contain raw lacquer, drying oils, proteins, polysaccharides, waxes, natural resins, mineral pigments, fillers, and later conservation materials. Long-term burial and aging processes, including oxidation, hydrolysis, cross-linking, microbial activity, and mineral contamination, may alter or obscure the original chemical signatures of these materials [4,5,6]. As a result, conventional microscopic and spectroscopic methods are valuable for revealing stratigraphy, morphology, pigments, and functional groups, but they are often insufficient for independently identifying degraded organic matrices, trace additives, and biological sources [7,8].

In this analytical context, the term “molecular recognition” requires clarification in lacquerware research. In this review, molecular recognition is used in a broad heritage-science sense to describe analytical strategies that identify lacquer materials through chemically or biologically specific markers. These approaches rely on different analytical principles. Pyrolysis gas chromatography/mass spectrometry (Py-GC/MS) enables the identification of lacquer matrices and additives through diagnostic pyrolysis fingerprints, including alkyl catechols, alkyl phenols, nitrogen-containing products, anhydrosugars, and long-chain aliphatic compounds [5,9,10,11]. In contrast, enzyme-linked immunosorbent assay (ELISA) and related glycoprotein-based assays depend on antigen–antibody interactions and are therefore better understood as biomolecular or immunological recognition methods for lacquer source differentiation [12,13]. High-performance liquid chromatography (HPLC) and sodium dodecyl sulfate polyacrylamide gel electrophoresis (SDS-PAGE) provide compositional profiling of phenolic and glycoprotein fractions, whereas expert systems do not perform molecular recognition directly but integrate morphological, elemental, spectroscopic, chromatographic, immunological, and historical datasets for cross-validation and conservation decision-making.

Therefore, this review distinguishes three complementary levels of evidence in lacquerware characterization: morphological and elemental observation, chemically specific molecular fingerprinting, and biomolecular source recognition. It first evaluates traditional microscopic, spectroscopic, and elemental techniques and their limitations in degraded lacquer systems. It then discusses recent progress in pyrolysis gas chromatography/mass spectrometry, immunological identification, and multidimensional expert systems. Finally, representative case studies are compared to show how cross-validation among stratigraphic, elemental, spectroscopic, chromatographic, immunological, and archaeological evidence can improve material identification, degradation assessment, conservation decision-making, and the reconstruction of historical lacquer craftsmanship.

Unlike previous reviews that mainly summarize analytical techniques for Chinese lacquer identification, this review focuses on the evidential logic of molecular recognition and cross-validation. It distinguishes chemically specific molecular fingerprinting, biomolecular source recognition, and multi-modal expert-system integration and further evaluates how these strategies support material identification, manufacturing reconstruction, degradation uncertainty assessment, and conservation decision-making. The novelty of this review lies in its diagnostic framework rather than in a simple inventory of analytical methods.

## 2. Traditional Analytical Techniques and Their Limitations

Chronological assessment of lacquerware coatings. The manufacture date of a lacquerware coating is not determined solely by archaeological context, although archaeological stratigraphy, typology, inscriptions, and historical documentation remain essential chronological references. Physical and chemical methods can provide complementary evidence, but their applicability depends strongly on the sampled material and preservation state. Radiocarbon dating can be applied to organic components associated with lacquerware, including wooden substrates, textile reinforcement layers, plant fibers, carbonaceous residues, and, in some cases, Asian lacquer films themselves. Recent studies have shown that accelerator mass spectrometry radiocarbon dating of lacquer layers is possible when appropriate pretreatment is used to remove exogenous carbon and conservation contaminants; however, direct dating of lacquer coatings remains complicated by mixed organic additives, later repairs, aged restoration materials, and burial contamination [14,15]. Dendrochronology may provide more precise chronological information for wooden substrates when sufficient growth-ring sequences are preserved, but it dates the wood rather than the lacquer application itself [16]. Luminescence-based methods, including thermoluminescence and optically stimulated luminescence, are generally more suitable for associated sediments, ceramics, heated minerals, or depositional contexts than for organic lacquer films, and therefore provide contextual rather than direct coating ages [17]. Chemical aging indicators, such as oxidation products, chain scission markers, changes in urushiol-derived pyrolysis products, and weakening of long-chain alkyl catechol signals, may help assess relative degradation states, but they cannot independently provide absolute manufacture dates because degradation is strongly affected by humidity, temperature, oxygen exposure, burial chemistry, microbial activity, and conservation history [5]. Therefore, lacquerware coating age should be evaluated through a combined chronological framework that integrates archaeological context, stratigraphy, substrate dating, radiocarbon evidence where feasible, and material-degradation indicators.

### 2.1. Microscopic Morphological Observation Techniques

Microscopic observations primarily provide structural and morphological information, including layer number, layer thickness, particle distribution, cracks, pores, and interfaces. However, microscopy alone cannot determine lacquer source, precise organic composition, or degradation chemistry. Long-term burial environments may induce oxidation, cross-linking, hydrolysis, microbial activity, and soil/mineral contamination, which can obscure original microstructural features and complicate interpretation [7,8]. Therefore, microscopic methods are indispensable for stratigraphic analysis but remain insufficient for molecular-level material identification, source attribution, and degradation assessment. Integration with FTIR, Raman spectroscopy, SEM-EDS, and Py-GC/MS is necessary to achieve comprehensive characterization of archaeological lacquer systems [1,5].

Ancient lacquerware generally employed a multi-layer composite application process, typically structured as: wood core → lacquer paste → primer → topcoat. By examining the cross-sectional morphology of lacquer fragments under microscopic magnification, researchers can directly acquire critical information regarding the number of lacquer layers, thickness distribution, and sequence of application.

Wu et al., 2022 conducted sectional microscopy studies on lacquer box fragments from six tombs [18]. Their analysis revealed that lacquer boxes excavated from the Han Dynasty tomb at Haiqu, Shandong, exhibited refined stratification characteristics, specifically: lacquer paste layer → primer layer → topcoat (colored lacquer and painted layer). These findings demonstrate the reliability of microscopic observation methods for identifying stratigraphic structures and lacquering sequences in ancient Chinese lacquerware.

**Figure 1:**
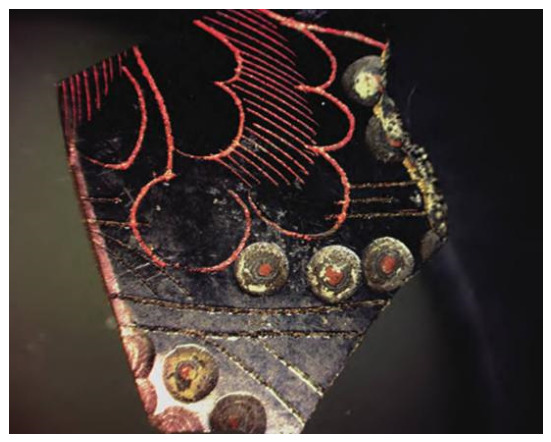
Southern Song lacquerware fragment with carved floral and filled-color rhinoceros pattern. Reprinted with permission from Ref. [7]. Copyright 2023.

Following advancements in optical instrumentation, high-resolution microscopy has enabled micro-destructive analysis of complex, multi-layered lacquer fragments, providing detailed morphological evidence of substrate structures and lacquering sequences [7,8]. For example, Southern Song Dynasty fragments with carved floral motifs and colored inlays were analyzed to reveal both layer architecture and particle size distribution in lacquer ash layers (Figure 1). Microscopic techniques remain limited in resolving chemical composition or trace organic additives in highly degraded matrices. They are affected by oxidation, cross-linking, hydrolysis, and soil/mineral contamination during long-term burial, highlighting the need for complementary analytical methods.

### 2.2. Basic Spectrum and Energy Spectrum Analysis

With microscopic morphological observations elucidating the multi-layered structure, fundamental spectroscopic techniques, including FTIR and Raman spectroscopy, combined with SEM-EDS, have been extensively applied to identify inorganic pigments and organic matrices in lacquer artifacts. These methodologies provide core compositional information at both elemental and functional-group levels, facilitating material characterization that is crucial for chronological classification, craft interpretation, and conservation decision-making.

Yang et al., 2021 confirmed cinnabar and carbon black as the main pigments in Han Dynasty lacquer films, with minimal tung oil in the matrix [19]. Hao et al., 2021 used combined micro-Raman and infrared spectroscopy to resolve 14 commonly used mineral pigments in lacquer artifacts from different historical periods, providing minimally invasive compositional insights [20]. Xiao et al., 2020 further demonstrated the potential of NIR spectroscopy combined with chemometric models for quantifying the dry oil/raw lacquer ratio in severely aged coatings, thereby overcoming some limitations of conventional FTIR-ATR [4].

However, traditional spectroscopic methods alone struggle to resolve complex aged organic mixtures, multi-component binders, and trace additives. In severely aged lacquer films, oxidation and cross-linking may broaden or shift FTIR absorption bands, while hydrolysis and burial contamination may introduce signals from soil minerals, carbonates, silicates, or absorbed moisture. These changes increase uncertainty in assigning original lacquer components, particularly when proteinaceous, polysaccharide, drying-oil, or resin additives coexist in the same layer [4,6]. Therefore, FTIR and Raman spectroscopy are most effective for functional-group screening and pigment identification, but they require Py-GC/MS, chemometric modeling, and microscopic cross-validation when applied to degraded archaeological lacquerware [20].

Traditional analytical techniques remain essential for the preliminary characterization of lacquerware, particularly for revealing stratigraphic structures, pigment distribution, elemental composition, and broad functional-group information. Microscopy provides direct evidence of layer number, thickness, particle distribution, cracks, pores, and interfaces, while Fourier transform infrared spectroscopy, Raman spectroscopy, and scanning electron microscopy–energy dispersive spectroscopy help identify pigments, fillers, and inorganic components. However, these methods alone are often insufficient for assigning lacquer sources, degraded organic matrices, trace additives, and complex multi-component binders. Oxidation, hydrolysis, cross-linking, microbial activity, and burial contamination may obscure original material signatures and increase interpretive uncertainty. Therefore, traditional methods should be regarded as foundational but not independently conclusive; reliable lacquerware characterization requires their integration with molecular-level and chemically specific analytical techniques.

## 3. Breakthroughs in Cutting-Edge Molecular Recognition and Chemically Specific Analytical Strategies

### 3.1. Py-GC/MS Analysis

Py-GC/MS has emerged as a pivotal analytical technique for characterizing organic matter in lacquerware artifacts. This approach employs high-energy thermal decomposition to rapidly cleave chemical bonds in non-volatile macromolecular organic compounds, generating smaller molecular fragments that retain characteristic parent structures. These fragments are subsequently separated via gas chromatography and detected by mass spectrometry, allowing qualitative and semi-quantitative molecular-level identification of organic constituents and additives.

Py-GC/MS offers several advantages over conventional chromatographic methods, including the elimination of complex pretreatment steps, high sensitivity, and minimal sample requirements at the microgram level [1,6]. It facilitates the detection of multi-component mixtures within lacquer films and lacquer dust, thereby meeting the requirements of micro-invasive analysis in cultural heritage conservation.

Asian lacquer, as a highly cross-linked natural polymer, can be identified through diagnostic pyrolysis products such as alkyl catechols, alkyl phenols, aliphatic aldehydes, and ketones. Studies on artificially aged lacquer polymers from different species have shown that adjusting pyrolysis temperatures and employing online derivatization techniques, such as silanization with hexamethyldisilazane (HMDS) or methylation with tetramethylammonium hydroxide (TMAH), can help distinguish primary cracking reactions from secondary products [9]. Full two-dimensional gas chromatography/mass spectrometry (Py-GC×GC/MS) further improves the resolution of complex mixtures by identifying marker compounds such as alkyl hydrocarbons, alkylbenzenes, alkylphenols, and alkyl catechols. This approach has enabled fingerprint differentiation of Chinese lacquer, Vietnamese lacquer, and Burmese lacquer, including the separation of regional isomers that are difficult to distinguish using conventional one-dimensional chromatography [21].

**Figure 2:**
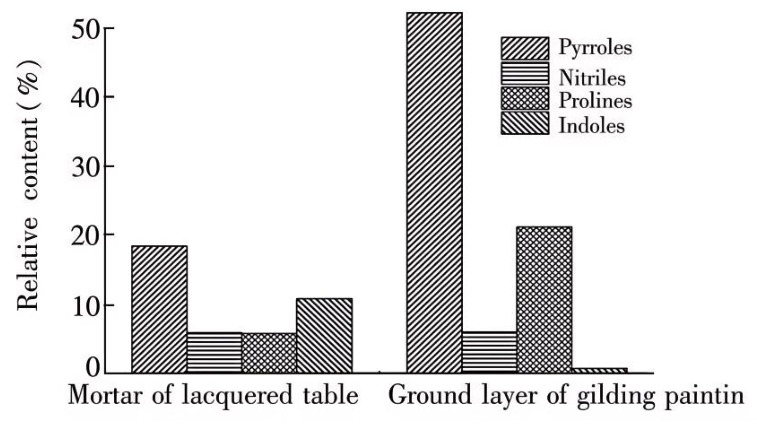
Relative distribution of nitrogen-containing pyrolysis products in proteinaceous binders from lacquerware samples. Redrawn by the authors based on [10]. Reprinted with permission from Ref. [10]. Copyright 2020 SCIENCE PRESS.

Beyond lacquer matrix identification, Py-GC/MS has enabled the characterization of complex additives incorporated into lacquerware, particularly proteinaceous, polysaccharide, and wax-based binders. For protein-based materials, including porcine blood, animal glue, and egg white, nitrogen-containing pyrolysis products such as pyrroles, nitriles, proline-related compounds, and indoles can serve as molecular markers for distinguishing adhesive types used in lacquerware ground layers (Figure 2) [10].

**Figure 3:**
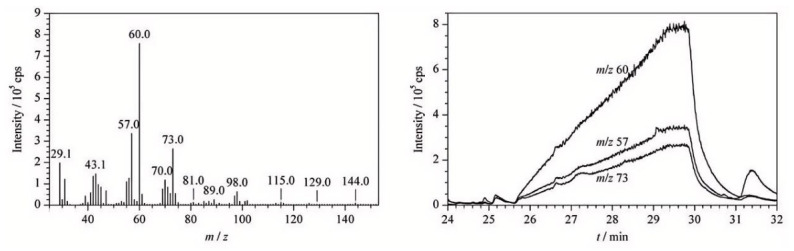
Mass spectrometric profile of 1,6-anhydro-β-D-glucose derived from starch pyrolysis, showing major fragment ions within the retention-time range of 25.2–31.0 min. Reprinted with permission from Ref. [11]. Copyright 2022 SCIENCE PRESS.

**Figure 4:**
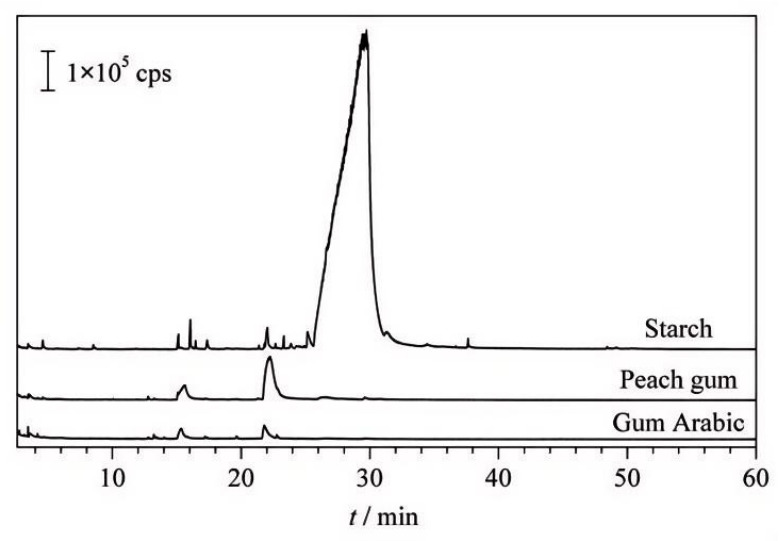
Extracted ion chromatograms at *m*/*z* 60 from Py-GC/MS analysis of starch, peach gum, and gum arabic, showing diagnostic ion-current patterns for differentiating polysaccharide binders. Reprinted with permission from Ref. [11]. Copyright 2022 SCIENCE PRESS.

For polysaccharide-based additives, such as starch, peach gum, and gum arabic, characteristic pyrolysis products including 1,6-dehydrated β-D-glucose, furfural, and hydroxymethylfurfural provide diagnostic evidence for identifying plant-derived binders. Characteristic ion patterns, such as *m*/*z* 60 and *m*/*z* 101, further support the screening and differentiation of polysaccharide adhesives within complex lacquer films (Figure 3 and Figure 4) [11].

Wax materials are characterized by homologous distributions of straight-chain alkanes, fatty acids, fatty alcohols, and esters. Beeswax generally exhibits strong odd-carbon-number alkane distributions and long-chain palmitate esters, whereas mineral waxes are dominated by saturated hydrocarbons with limited oxygenated products [10].

From a chemical perspective, the diagnostic capability of Py-GC/MS depends on the systematic interpretation of marker classes and representative compounds rather than isolated compounds (Table 1). For Asian lacquers, alkyl catechols and alkyl phenols are regarded as key fingerprints of urushiol- or laccol-type polymers, while alkylbenzenes and long-chain hydrocarbons provide supplementary evidence for lacquer tree species differentiation and degradation assessment [9,21]. Chinese lacquer derived from *Toxicodendron vernicifluum* is typically characterized by C15 side-chain catechols and phenols, whereas Vietnamese and Burmese lacquers contain laccol- and thitsiol-related derivatives with distinct alkyl substitution patterns. In aged lacquer films, oxidation and cross-linking reactions frequently lead to the formation of aldehydes, ketones, and low-molecular-weight aromatic compounds, which can be used as indicators of degradation state and burial-induced alteration.

These systematically organized marker classes provide a chemically specific framework for interpreting Py-GC/MS data, enabling more reliable differentiation of lacquer matrices, organic additives, and degradation products in archaeological lacquerware. Aging and burial conditions must be considered when interpreting Py-GC/MS results. Oxidation and cross-linking can decrease the abundance of long-chain alkyl catechols and unsaturated side-chain derivatives, while hydrolysis may generate secondary fatty acids, aldehydes, ketones, and low-molecular aromatic compounds. Soil and mineral contamination may also suppress weak organic signals or introduce exogenous compounds. Consequently, the absence or weakening of diagnostic markers should not be interpreted as definitive evidence for the absence of a specific lacquer source or additive. Instead, Py-GC/MS results should be evaluated together with stratigraphic, elemental, spectroscopic, and archaeological context [5,9,22].

Overall, Py-GC/MS is not limited to general lacquer matrix identification but extends to molecular-level characterization of complex, multi-component systems in lacquerware. The integration of pyrolysis, derivatization, and mass spectrometric analysis enables the simultaneous identification of lacquer matrices, organic additives, waxes, and degradation products, thereby providing critical evidence for reconstructing ancient material formulation, craftsmanship, and conservation interventions.

### 3.2. Immunological Identification Techniques Based on Glycoproteins

While chromatography and mass spectrometry methods have advanced the analysis of organic small molecules and polymer frameworks in lacquer films, precise differentiation of closely related biological materials remains a persistent challenge in archaeological science. Natural lacquer saps from Asia are derived from three primary lacquer tree species—Toxicodendron vernicifluum, Toxicodendron succedaneum, and Melanorrhoea usitata—with T. vernicifluum in China having been subject to millennia of artificial cultivation and selection [13]. Accurately identifying whether ancient lacquerware employed wild or domesticated lacquer is essential for reconstructing historical breeding practices and lacquering technologies, yet traditional physicochemical analyses often cannot resolve differences at the species or cultivar level.

In contrast, immunological identification techniques that target macromolecular biomarkers, such as lacquer glycoproteins, provide a complementary approach to biological source attribution. Lacquer sap contains not only low-molecular-weight phenolic compounds but also high-molecular-weight glycoproteins involved in catalytic polymerization and film formation [23,24]. By targeting these macromolecular constituents, biochemical assays may generate species- or cultivar-related biomolecular profiles.

However, such results should be interpreted as supportive rather than absolute evidence of source identification. The reliability of glycoprotein-based assays depends strongly on protein preservation, extraction efficiency, antibody specificity, and possible cross-reactivity among related lacquer species or degraded proteinaceous materials [12,25,26]. In archaeological lacquerware, long-term oxidation, hydrolysis, microbial degradation, and mineral contamination may damage epitopes or reduce antibody binding efficiency, thereby increasing the risk of false-negative or ambiguous results.

Therefore, ELISA, HPLC, and SDS-PAGE are most useful when used as complementary tools alongside Py-GC/MS, FTIR, Raman spectroscopy, and SEM-EDS. Rather than independently confirming lacquer provenance, they provide biomolecular evidence that can strengthen source attribution when cross-validated with chemical fingerprints and archaeological context [12,13].

### 3.3. Multidimensional Expert System for “Morphology–Composition–Structure–Source”

As analytical technologies for lacquerware become increasingly diversified, single detection methods no longer satisfy the comprehensive extraction needs of complex cultural heritage data. Lacquerware is inherently a multi-layered, multi-component composite material, which necessitates the development of a multidimensional expert system that integrates physical morphology, elemental composition, molecular structure, and biological source information. Such systems represent a leading trend in cultural heritage science, aiming to establish a holistic cognitive framework that bridges microscopic morphology observation with macroscopic craftsmanship interpretation. The core logic of this expert system lies in coupling multiple micro-destructive and non-destructive analytical techniques to systematically interpret the “full information” profile of lacquerware artifacts. Accurate micro-sampling and analysis protocols form the foundation of this process, enabling reliable acquisition of multi-modal datasets. In practice, researchers have successfully integrated advanced microscopic imaging, elemental analysis (SEM-EDS), vibrational spectroscopies (micro-Raman and FTIR), and pyrolytic mass spectrometry (Py-GC/MS) to generate complementary datasets that span structural, elemental, and molecular dimensions (e.g., micro-area studies on Qing Dynasty lacquer objects) [27].

After obtaining individual datasets, multi-dimensional expert systems employ cross-validation of heterogeneous experimental results to establish standardized micro-loss analysis workflows for complex lacquer matrices. This procedural integration effectively defines an expert review framework in which stratigraphic morphological analysis informs compositional determinations, and compositional characteristics guide technological and provenance inferences. Such frameworks provide standardized technical support for both conservation/restoration decision-making and craft research [27,28].

The multidimensional system excels at resolving the long-standing disconnect between an artifact’s “appearance” and its material “essence.” By aligning stratification patterns observed by microscopic morphology with elemental distribution data from SEM-EDS, researchers can accurately map pigment and filler distribution patterns within lacquer films [28]. Meanwhile, organic molecular fingerprints derived from Py-GC/MS and related techniques provide deeper insights into binder composition, organic additive profiles, and degradation products, significantly enhancing the accuracy of component identification [29].

Beyond compositional identification, expert systems increasingly leverage source attribution analysis, integrating high-resolution molecular data with historical, geographical, and material culture records to deduce production origins, quality grades, and regional craft traditions. For example, multimodal micro-area studies have reconstructed engineered lacquer stratigraphy in Qing Dynasty artifacts by identifying drying oils, proteins, starches, and resins through combined microscopy, SEM-EDS, micro-Raman, and THM-Py-GC/MS [30].

The ongoing development of multidimensional expert systems also relies on the construction of large-scale spectral and mass spectrometry databases, which consolidate characteristic peaks and profiles for mineral pigments, proteins, polysaccharides, waxes, and other additive classes. These enriched knowledge bases form the backbone of data-driven expert systems, enabling authentication and diagnostic workflows that transcend reliance on subjective expert experience. By leveraging objective multidimensional data matrices, such systems facilitate the digital reconstruction of lacquerware production techniques and provide scientifically grounded evaluations of material quality and craftsmanship. This transition—from isolated instrument applications to systematic analytical frameworks—marks a new phase in lacquerware cultural heritage analysis, paving the way toward “smart cultural preservation.”

Section summary: Cutting-edge molecular and biomolecular recognition strategies provide more specific evidence for identifying lacquer matrices, organic additives, degradation products, and biological sources. Pyrolysis gas chromatography/mass spectrometry is particularly valuable because it detects diagnostic marker classes, including alkyl catechols, alkyl phenols, nitrogen-containing pyrolysis products, anhydrosugars, long-chain aliphatics, aldehydes, and ketones. These markers support the differentiation of lacquer types, proteinaceous binders, polysaccharide adhesives, waxes, and degradation states. Immunological identification based on lacquer glycoproteins further complements chemical fingerprinting by offering potential evidence for species- or cultivar-level source attribution. Nevertheless, both approaches require cautious interpretation. Pyrolysis markers may be weakened or altered by aging and burial conditions, while immunological assays depend on protein preservation, extraction efficiency, antibody specificity, and possible cross-reactivity. Thus, molecular recognition methods are most reliable when interpreted together with stratigraphic, elemental, spectroscopic, and archaeological evidence.

## 4. Case Analysis of Technology Application

To illustrate the practical application of modern analytical technologies in lacquerware research, three recent studies were examined, each employing a combination of microscopic, spectroscopic, and chromatographic techniques to characterize complex material systems.

### 4.1. Case 1: Analysis of a Lacquered Pipe from 16th–17th Century Sweden

**Figure 5:**
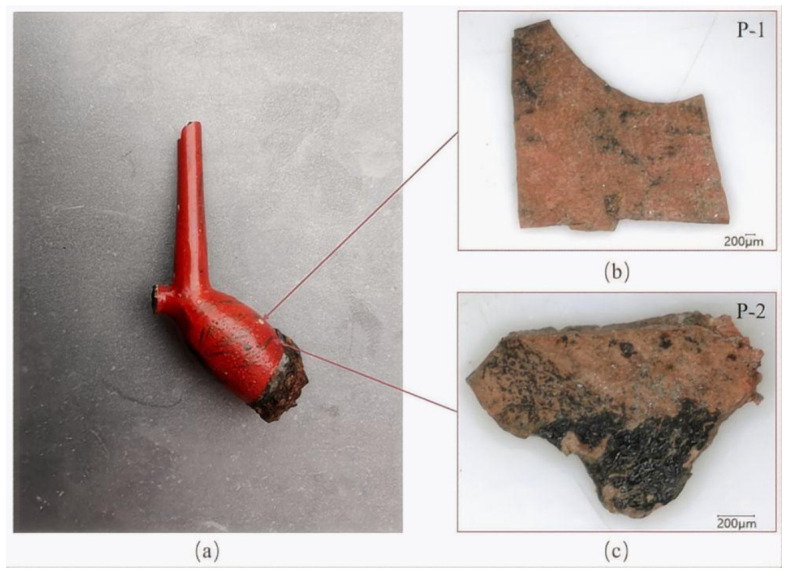
Photographs and microscopic fragments of the Swedish archaeological lacquer pipe: (**a**) the complete pipe, (**b**) red fragment P-1, and (**c**) black fragment P-2. Reprinted from Ref. [3].

This case study focuses on the scientific analysis of a lacquered pipe excavated from a seventeenth-century archaeological site in Sweden, initially presumed to be imported Chinese lacquerware due to the European admiration for Asian lacquer during the sixteenth to eighteenth centuries [3]. To accurately determine its composition, a multi-analytical approach was applied, combining attenuated total reflectance Fourier transform infrared spectroscopy (ATR-FTIR), thermally assisted hydrolysis and methylation pyrolysis gas chromatography/mass spectrometry (THM-Py-GC/MS), SEM-EDS, and Raman spectroscopy. Small samples were taken from both red and black areas of the pipe (labeled as P-1 and P-2, see Figure 5) for detailed material characterization [3].

The ATR-FTIR spectra indicated broad hydroxyl peaks and ester carbonyl signals consistent with resinous materials, suggesting a coating more akin to European shellac than Asian lacquer. THM-Py-GC/MS analysis confirmed shellac as the principal organic matrix, accompanied by pine resin and turpentine as additives, with characteristic pyrolytic products such as aleuritic acid methyl esters and laccijalaric acid derivatives. SEM-EDS and Raman spectroscopy identified the pigments: cinnabar in the red layer and carbon-based black in the black layer. This multi-technique workflow allowed precise identification of both organic binders and inorganic pigments, as well as the determination of layer stratigraphy, with the red coating approximately 60 µm thick and the black bamboo pattern overlay 240 µm [3].

From a technical perspective, this case illustrates the value of integrated analytical approaches for artifact authentication and cultural heritage studies. The combination of Py-GC/MS for molecular fingerprinting, ATR-FTIR for functional group analysis, and SEM-EDS/Raman spectroscopy for pigment identification provides a comprehensive view of material composition and construction methods. Furthermore, by comparing the identified components with historical records of European lacquer production, the study highlighted the use of locally sourced shellac in imitation of Chinese lacquer, demonstrating both technological adaptation and cultural exchange [29,30].

Across this case, a multi-analytical scientific workflow effectively revealed both the material composition and historical context of the artifact, demonstrating how contemporary technology supports the authentication, conservation, and historical interpretation of lacquerware [21,31].

### 4.2. Case 2: Structural and Compositional Characterization of a Lacquered Ear Cup from the Nanyue Kingdom

This case study examines a lacquered ear cup excavated from the Luobowan tomb complex in Guigang, Guangxi, attributed to the Nanyue Kingdom of the early Han dynasty. The study employed a comprehensive multi-analytical approach including optical microscopy (OM), SEM-EDS, XRD, FT-IR, and Py-GC-MS to investigate the cross-sectional structure, material composition, and preservation state of the artifact [32].

**Figure 6:**
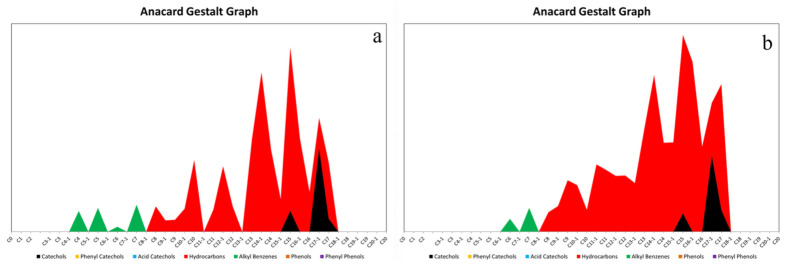
Py-GC-MS analysis results of the lacquer films from the lacquered ear cup: (**a**) red lacquer film; (**b**) black lacquer film [32].

The cross-sectional analysis revealed a four-layered structure: a wooden core, a fabric reinforcement layer, a lacquer ground layer, and a lacquer film layer, reflecting the technical practices of Central Plains Han dynasty lacquerware [32]. The wooden core was identified as *Phoebe* sp. (Lauraceae), the fabric layer likely hemp, and the lacquer ground composed of natural Chinese lacquer mixed with SiO_2_ from brick or tile powder. Notably, the lacquer films were a blend of Chinese urushi and Vietnamese laccol, with cinnabar and carbon black used as red and black pigments, respectively (Figure 6). The stratigraphy, composition, and pigment selection demonstrate a high degree of craftsmanship and regional adaptation within Nanyue lacquer production [9,33,34,35].

The Py-GC-MS analysis enabled identification of the C17-structured urushiol compounds in the red lacquer and C15-structured laccol compounds in the black lacquer. FT-IR analysis further revealed differences in organic structures, with the red lacquer exhibiting strong C–H stretching vibrations and prominent carbonyl absorption bands, whereas the black lacquer showed higher moisture content and more porous microstructure, likely due to pigment properties and aging processes [32].

This case illustrates the integration of chemical, structural, and microscopic analyses for reconstructing historical lacquer technology. By combining Py-GC-MS, SEM-EDS, and FT-IR data, the study identified both organic lacquer types and inorganic pigments, enabling insights into cross-regional material exchanges, technological adaptation, and craftsmanship standards during the Nanyue Kingdom period [32,33,34,35]. Such integrated methodologies provide critical reference for conservation planning and highlight the importance of multi-analytical approaches in cultural heritage science.

### 4.3. Case 3: Molecular Fingerprinting of a Tang Dynasty Lacquered Leather Artifact

This case study investigates a Tang Dynasty lacquered leather artifact, recovered from Jiaozuo, Henan Province. Due to the severe deterioration of the organic material, the study applied an integrated analytical workflow combining FTIR, SEM-EDS, XRD, and Py-GC–MS to identify both the leather substrate and the lacquer coating [23]. The artifact fragments, along with surrounding soil, were sampled to capture residual fibers and inorganic mineral inclusions. SEM-EDS analysis revealed the presence of C, N, O, Ca, Al, and Si, indicating both the leather fibers and burial environment residues [23]. XRD confirmed the mineral composition of quartz, calcite, and albite, consistent with sedimentary soil contamination. FTIR spectra displayed broad peaks corresponding to hydroxyl and amino groups from collagen, as well as characteristic vibrations of catechols from lacquer.

**Figure 7:**
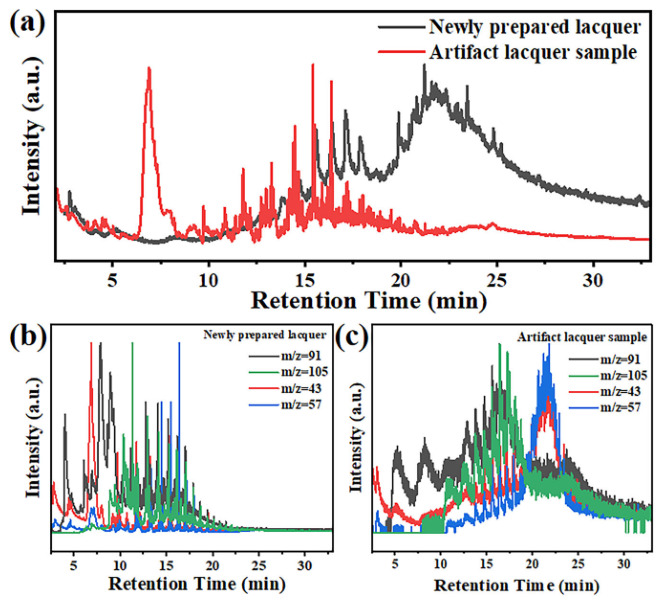
Py-GC–MS analysis of artifact lacquer sample and newly prepared lacquer. (**a**) TIC comparison; (**b**) EIC of artifact lacquer sample; (**c**) EIC of newly prepared lacquer. Signals at *m*/*z* = 43, 57, 91, 105 highlight alkanes and alkylbenzenes [23].

Py-GC–MS provided detailed molecular fingerprinting of the lacquer component. The total ion chromatogram (TIC) was divided into two zones: Zone I containing aliphatic aldehydes and short unsaturated hydrocarbons, and Zone II with long-chain alkanes and alkyl-substituted benzenes. Notably, Figure 7 compares the TICs and extracted ion chromatograms (EIC) of the artifact and a newly prepared reference lacquer. The artifact exhibited weaker alkane chain signals in Zones II, reflecting degradation of long-chain urushiol derivatives due to aging, while the newly prepared lacquer displayed stronger and more stable signals, indicating intact crosslinked urushiol structures. These analyses confirm that the artifact contains urushiol-based lacquer, likely sourced from Toxicodendron vernicifluum, consistent with historical Chinese lacquer usage.

This case demonstrates the effectiveness of molecular fingerprinting in reconstructing severely deteriorated organic artifacts. By integrating microscopic, elemental, and molecular analyses, researchers can identify raw lacquer type, reconstruct manufacturing techniques, and assess degradation mechanisms [36,37]. Furthermore, such comprehensive analytical workflows provide critical insights for conservation strategies, enabling informed decisions about preservation, restoration, and authentication of Tang Dynasty lacquered leather artifacts.

### 4.4. Comparative Analysis of the Three Cases

The three case studies demonstrate that integrated analytical workflows are essential for distinguishing material identity, technological origin, and degradation-related uncertainty in lacquerware research. In the Swedish pipe, ATR-FTIR, THM-Py-GC/MS, SEM-EDS, and Raman spectroscopy showed that the coating was not Asian lacquer but a European shellac-based imitation. The key diagnostic evidence included shellac-related pyrolysis markers, such as aleuritic acid methyl esters and laccijalaric acid derivatives, together with cinnabar and carbon black pigment identification [3]. In the Nanyue ear cup, cross-sectional microscopy and Py-GC-MS revealed a multi-layered structure and a differentiated color-layer distribution: the red lacquer film contained C17-structured urushiol-related compounds, whereas the black lacquer film contained C15-structured laccol-related compounds, suggesting the combined use of Chinese urushi and Vietnamese laccol within a regional production system [32]. In the Tang lacquered leather artifact, Py-GC-MS identified urushiol-based lacquer through catechol derivatives, aliphatic aldehydes, short-chain hydrocarbons, long-chain alkanes, and alkyl-substituted benzenes; however, weaker alkane-chain signals should not be attributed only to raw-material differences, because oxidation, hydrolysis, burial contamination, and marker suppression during long-term aging may also reduce the abundance of long-chain urushiol-derived products.

Overall, these cases show that Py-GC/MS is indispensable for molecular-level identification of organic lacquer matrices, additives, and degradation products (Table 2). Spectroscopy and microscopy provide essential evidence for layer structure, pigments, and inorganic components, but reliable interpretation of degraded lacquerware requires cross-validation among stratigraphic, elemental, spectroscopic, chromatographic, and historical evidence.

Section summary: The three representative case studies demonstrate that lacquerware interpretation is most reliable when multiple lines of evidence are combined rather than when a single technique is used independently. The seventeenth-century Swedish lacquered pipe, the Nanyue Kingdom lacquered ear cup, and the Tang Dynasty lacquered leather artifact each show different analytical challenges, including complex stratigraphy, mixed organic and inorganic materials, degradation of molecular markers, burial contamination, and uncertainty in source attribution. Across these cases, microscopy and elemental analysis clarify layered structures and inorganic components, vibrational spectroscopy provides functional-group and pigment information, and pyrolysis gas chromatography/mass spectrometry offers molecular fingerprints of lacquer matrices and additives. The common methodological lesson is that cross-validation among morphological, elemental, spectroscopic, chromatographic, and archaeological evidence can reduce uncertainty, improve material identification, and support more reliable reconstruction of manufacturing techniques and conservation histories.

## 5. Conclusions

This review demonstrates that the characterization of Chinese lacquerware requires a hierarchical and cross-validated analytical framework rather than reliance on any single technique. First, microscopy and scanning electron microscopy–energy dispersive spectroscopy are effective for revealing stratigraphy, pigment distribution, fillers, cracks, pores, and inorganic components, while Fourier transform infrared spectroscopy and Raman spectroscopy provide useful information on functional groups and mineral pigments. However, these methods are limited when applied to severely degraded lacquer films because oxidation, hydrolysis, cross-linking, microbial activity, and burial contamination may obscure original chemical signals and complicate source attribution.

Second, pyrolysis gas chromatography/mass spectrometry provides molecular-level evidence for identifying lacquer matrices, organic additives, and degradation products. Its diagnostic value depends on the systematic interpretation of marker classes, including alkyl catechols, alkyl phenols, nitrogen-containing pyrolysis products, anhydrosugars, long-chain aliphatics, aldehydes, and ketones. Third, immunological and glycoprotein-based assays can support lacquer source differentiation, but their reliability is constrained by protein preservation, extraction efficiency, antibody specificity, and possible cross-reactivity. Finally, the three representative case studies confirm that robust interpretation depends on cross-validation among morphological, elemental, spectroscopic, chromatographic, immunological, and archaeological evidence. Integrated multi-analytical workflows therefore provide more reliable support for material identification, degradation assessment, conservation decision-making, and reconstruction of historical lacquer craftsmanship.

## Figures and Tables

**Table 1:** Diagnostic pyrolysis marker classes and representative compounds identified in archaeological lacquerware.

Material Type	Marker Class	Representative Compounds	Analytical Significance
Asian lacquer matrix	Alkyl catechols; alkyl phenols	C15 urushiol derivatives; C17 laccol derivatives; alkyl-substituted phenols	Identification of urushiol-, laccol-, or thitsiol-type lacquer
Degraded lacquer	Aldehydes; ketones; low-molecular aromatic compounds	Aliphatic aldehydes; alkylbenzenes; phenolic fragments	Assessment of oxidation, cross-linking, and burial-induced degradation
Proteinaceous binders	Nitrogen-containing pyrolysis products	Pyrroles; nitriles; indoles; proline-related compounds	Identification of animal glue, porcine blood, egg white, or protein additives
Polysaccharide binders	Anhydrosugars; furan derivatives	Levoglucosan; furfural; hydroxymethylfurfural	Detection of starch, flour paste, peach gum, gum arabic, or plant gums
Waxes	Long-chain aliphatics; esters	Alkanes; fatty acids; fatty alcohols; palmitate esters	Differentiation of beeswax, palm wax, mineral wax, or conservation waxes

**Table 2:** Comparative analytical outcomes and uncertainties of three representative lacquerware case studies.

Case Study	Key Techniques	Diagnostic Evidence	Main Interpretation	Uncertainty/Limitation
Seventeenth-century Swedish lacquered pipe	ATR-FTIR; THM-Py-GC/MS; SEM-EDS; Raman spectroscopy	Shellac-related pyrolysis markers, including aleuritic acid methyl esters and laccijalaric acid derivatives; cinnabar and carbon black pigment distribution	Identification of a European shellac-based imitation rather than Asian lacquer; reconstruction of a resinous layered coating system	Shellac-like resin signals require distinction from aged resinous materials, conservation residues, or later contamination; interpretation should be cross-checked with stratigraphy and historical context
Nanyue Kingdom lacquered ear cup	Cross-sectional microscopy; Raman spectroscopy; FTIR; Py-GC-MS	Multi-layered structure; red lacquer film with C17 urushiol-related compounds; black lacquer film with C15 laccol-related compounds; pigment stratigraphy	Reconstruction of multilayer lacquering technology and evidence for combined use of Chinese urushi and Vietnamese laccol in colored decorative layers	Spectral overlap, layer mixing, aging, and pigment effects may increase uncertainty in layer-specific source attribution
Tang Dynasty lacquered leather artifact	Py-GC-MS; FTIR; SEM-EDS; molecular fingerprint analysis	Catechol derivatives; aliphatic aldehydes; short-chain hydrocarbons; long-chain alkanes; alkyl-substituted benzenes; weaker alkane-chain signals	Identification of urushiol-based lacquer on a degraded leather substrate; interpretation of composite organic formulation and lacquered leather technology	Weaker alkane-chain signals may result from oxidation, hydrolysis, burial contamination, or marker suppression, not only from raw-material differences

## Data Availability

The original contributions presented in this study are included in the article. Further inquiries can be directed to the corresponding author.
